# Does regional compared to local anaesthesia influence outcome after arteriovenous fistula creation?

**DOI:** 10.1186/1745-6215-14-263

**Published:** 2013-08-19

**Authors:** Alan James Robert Macfarlane, Rachel Joyce Kearns, Emma Aitken, John Kinsella, Marc James Clancy

**Affiliations:** 1 Department of Anaesthesia, Glasgow Royal Infirmary, 91 Wishart Street, Glasgow, Scotland G31 2HT, UK; 2 Renal Surgery/Transplant Unit, Western Infirmary, Dumbarton Road, Glasgow Scotland G11 6NT, UK; 3 Academic Unit of Anaesthesia Unit, Pain & Critical Care Medicine 4th Floor, Walton Building, Glasgow Royal Infirmary, 91 Wishart Street, Glasgow, Scotland G31 2HT, UK

**Keywords:** Fistula, Patency, Flow, Anaesthetic, Local, Nerve block, Renal failure

## Abstract

**Background:**

An arteriovenous fistula is the optimal form of vascular access in patients with end-stage renal failure requiring haemodialysis. Unfortunately, approximately one-third of fistulae fail at an early stage. Different anaesthetic techniques can influence factors associated with fistula success, such as intraoperative blood flow and venous diameter. A regional anaesthetic brachial plexus block results in vasodilatation and improved short- and long-term fistula flow compared to the infiltration of local anaesthetic alone. This, however, has not yet been shown in a large trial to influence long-term fistula patency, the ultimate clinical measure of success.

The aim of this study is to compare whether a regional anaesthetic block, compared to local anaesthetic infiltration, can improve long-term fistula patency.

**Methods:**

This study is an observer-blinded, randomised controlled trial. Patients scheduled to undergo creation of either brachial or radial arteriovenous fistulae will receive a study information sheet, and consent will be obtained in keeping with the Declaration of Helsinki. Patients will be randomised to receive either: (i) an ultrasound guided brachial plexus block using lignocaine with adrenaline and levobupivicaine, or (ii) local anaesthetic infiltration with lignocaine and levobupivicaine.

A total of 126 patients will be recruited. The primary outcome is fistula primary patency at three months. Secondary outcomes include primary patency at 1 and 12 months, secondary patency and fistula flow at 1, 3 and 12 months, flow on first haemodialysis, procedural pain, patient satisfaction, change in cephalic vein diameter pre- and post-anaesthetic, change in radial or brachial artery flow pre- and post-anaesthetic, alteration of the surgical plan after anaesthesia as guided by vascular mapping with ultrasound, and fistula infection requiring antibiotics.

**Conclusions:**

No large randomised controlled trial has examined the influence of brachial plexus block compared with local anaesthetic infiltration on the long-term patency of arteriovenous fistulae. If the performance of brachial plexus block increases fistulae patency, this will have significant clinical and financial benefits as the number of patients able to commence haemodialysis when planned should increase, and the number of “redo” or revision procedures should be reduced.

**Trial registration:**

This study has been approved by the West of Scotland Research Ethics Committee 5 (reference no. 12/WS/0199) and is registered with the ClinicalTrials.gov database (reference no. NCT01706354).

## Background

When chronic kidney disease (CKD) progresses to irreversible end stage renal failure, renal replacement therapy (RRT) is necessary for survival [1]. Haemodialysis (HD) is the commonest form of RRT. Good quality, stable vascular access is a major factor in determining survival in this group of CKD patients, and surgical creation of an arteriovenous fistula (AVF) is recommended as the optimal technique [2,3]. Unfortunately, approximately one-third of arteriovenous fistulae fail at an early stage [4]. This failure rate is influenced by both the pre-operative arterial and venous diameters and post-operative flow through the AVF, as well as a number of other patient and surgical factors [5,6]. Some anaesthetic techniques can directly influence venous diameter as well as intra- and post-operative blood flow [5], but there is no conclusive evidence as yet that any particular anaesthetic technique can significantly influence long term surgical outcome.

General anaesthesia, regional anaesthesia and local anaesthetic (LA) infiltration are all acceptable anaesthetic techniques for AVF creation. Regional anaesthesia, such as a brachial plexus block (BPB), involves injection of LA around nerves to specifically ‘block’ the motor and sensory nerves that supply the operative site, avoiding the need for general anaesthesia. Whilst general anaesthesia increases intra-operative vasodilatation, CKD patients are known to be at increased risk of peri- and post-operative complications [7,8]. Many of these complications can be avoided if regional or local anaesthesia are employed. Only regional anaesthesia, however, produces an associated sympathetic nerve block which results in an increased intraoperative venous diameter and vessel flow, both intra-operatively and, for several hours, post-operatively [9,10]. Maintenance of adequate blood flow through the fistula post-operatively can help prevent thrombosis and fistula failure and is important in fistula maturation [10]. Furthermore, arterial and venous spasm reduces flow and is more common with local infiltration than regional (or general) anaesthesia [11]. Several non-randomised trials have already demonstrated that, compared to local infiltration, a BPB results in lower immediate AVF failure rates [6,12] and also an improved surgical ability to identify the optimal graft site [13]. The effects, however, of these short-term benefits of regional anaesthesia on long-term AVF survival (patency) remain uncertain. To date there has been no large-scale randomised clinical trial examining this question. Recently it has been demonstrated that a BPB compared to LA alone significantly increased flow through the fistula up until eight weeks after surgery, but this did not translate into any difference in fistula patency [14]. Increased flow is important, but ultimately it is the patency of the AVF that is the major determinant of success. This trial was underpowered and the authors’ final conclusion was that larger scale clinical trials were required.

Anaesthetic technique, therefore, has the potential to modify a number of factors which may influence fistula success. We wish to investigate whether anaesthetic technique can influence fistula patency. Given the higher risks of general anaesthesia in these patients, we wish to compare BPB and LA techniques in an adequately powered trial. We hypothesise that the regional anaesthetic technique of an ultrasound guided BPB will, as a result of improved vasodilation and blood flow at the time of and shortly after surgery, increase the AVF patency rate at three months compared to those undergoing the procedure with LA infiltration. If this is the case, the number of patients able to commence HD when planned should be increased and the number of “redo” procedures should be reduced. This has clear financial implications as well as reducing inconvenience for patients.

## Methods

### Overview

This is a single centre, observer-blinded, randomised controlled trial. This study has been approved by the West of Scotland Research Ethics Committee 5 (reference no. 12/WS/0199) and is registered with the ClinicalTrials.gov database (reference no. NCT01706354). This study will be performed in keeping with the requirements of the Declaration of Helsinki.

### Hypothesis

The creation of arteriovenous fistulae under ultrasound guided brachial plexus block anaesthesia will result in an increased primary patency rate at three months compared to the use of local anaesthetic infiltration.

### Objectives and outcome measures

This study aims to compare local anaesthetic infiltration to a BPB anaesthetic technique for the surgical creation of an AVF, assessing in particular whether an increase in flow at the time of surgery with a BPB alters long-term outcome. The primary outcome measure is AVF primary patency at three months. Primary patency is defined as the unequivocal clinical presence of a thrill or audible bruit indicating flow through the fistula, in a fistula which has not required intervention to maintain or re-establish flow for whatever reason. Secondary outcomes include immediate post-operative patency (within one hour of termination of the procedure), primary patency at one month and one year, primary functional patency at three months and one year (that is, primary patency as described previously but which is also able to deliver flow greater than 350 ml/minutes without recirculation for the duration of haemodialysis), secondary patency at one year (that is, patency maintained by other therapeutic interventions, for example, interventional radiology), flow rates through AVF at one month, three months and one year, flow rates of the radial/brachial artery at one month, three months and one year measured by ultrasound, flow obtained at first HD, flow on HD after one year, change in cephalic vein diameter pre-/post-anaesthetic, change in radial or brachial artery flow pre-/post-anaesthetic, alteration of the surgical plan after anaesthetic and then venous/arterial mapping with ultrasound, AVF infection requiring antibiotics, pain during the procedure, pain one hour post-operatively, patient satisfaction, BPB success, complications including the requirement for conversion to general anaesthesia, the requirement for additional sedation or analgesics peri-operatively, post-operative paraesthesia or weakness suggestive of nerve damage, pneumothorax and time to perform the procedure.

### Study centre

Our centre is a tertiary referral facility for vascular access formation performing around 400 AVF creations per year. The necessary volume of clinical cases, presence of clinical expertise and equipment required for this study is well established in this unit. There is a wealth of experience on the use of ultrasound guidance for regional anaesthetic techniques within the department [15-18].

### Patients and enrolment

Patients scheduled to undergo primary AVF creation will be invited to participate in the study during their pre-assessment clinic visit staffed by the surgeon and the pre-assessment anaesthetic nurse. Inclusion criteria are English-speaking adults aged 18 to 85 who are competent to give consent scheduled for primary AVF formation at either the radial or brachial artery. Exclusion criteria are allergy to local anaesthetic, coagulopathy, infection at the anaesthetic or surgical site, patient preference for general or alternative anaesthesia, significant peripheral neuropathy or neurologic disorder affecting the upper extremity, pregnancy, previous AVF creation on the ipsilateral arm, known cephalic vein occlusion, central vein stenosis, brachial or radial artery stenosis and vein or artery less than 1.8 mm, as measured by ultrasound.

## Consent

The process of consent will be in accordance with the Declaration of Helsinki. All suitable patients will be fully informed that they are being asked to participate in a research study. The procedures involved in the study, and the chances of being assigned randomly to one of two groups, will be explained in person and via an information sheet approved by the West of Scotland Ethics Committee. A signed consent form will be obtained from each patient and retained by the investigators. Patients will be made aware of their right to withdraw from the study at any time without adverse effects on their clinical care.

### Pre-operative management

At room temperature, in a supine position and without any flexing of the elbow, the internal diameter of the cephalic vein in all patients will be measured by a blinded observer using a linear ultrasound probe at the wrist and elbow. The internal diameter of the basilic vein at the elbow will also be measured. In all cases, minimal compression will be applied with the probe and the vein measured in its most circular shape. To reduce intra-observer variability, the average of three measurements will be recorded for each parameter. Each vein measurement position will be marked on the skin. Flow through the radial artery or brachial artery, depending on which is being used for the AVF, will also be measured using Doppler ultrasound. In order to calculate flow, both vessel cross sectional area and time averaged velocity need to be measured. Cross sectional area is calculated by the machine after using ultrasound to measure vessel diameter. Time averaged velocity is measured in the long access with Doppler ultrasound maintaining an angle of insonation of less than 60 degrees. Using these figures, flow can then be calculated [19]. Again, the point of measurement will be marked on the skin and an average of three measurements will be recorded to reduce intra-observer variability.

### Randomisation

A computer generated 1:1 allocation sequence (in random permuted blocks of either four or six) will be created by an independent operator who is not directly involved with the study. Allocation concealment will be achieved using sequentially numbered, sealed opaque envelopes. An intravenous cannula will be inserted and standard monitoring applied, and then based on the randomization, the anaesthetist will perform either an ultrasound guided BPB as necessary or ask the operating surgeon to undertake local anaesthetic infiltration.

Patients in the Ultrasound Guided BPB group will receive an ultrasound guided brachial plexus block. A supraclavicular block will be performed unless there is a contraindication, in which case an axillary block will be used. A 1:1 mixture of 0.5% L-bupivicaine and 1.5% lignocaine with adrenaline (1 in 200,000) will be injected, using a minimum of 25 mls and up to a volume of 40 ml [20]. Maximum dose limits of 2 mg/kg for bupivicaine and 7 mg/kg for lignocaine with adrenaline will be observed, recognising that these are additive. The time taken to perform the block and any technical problems during block insertion will be recorded in the patient’s case report file (intravascular puncture, paraesthesia). Measurements of the sensory block of the musculocutaneous, median, radial and ulnar nerves will be recorded every five minutes by a non-blinded observer using a previously validated 3-point scale using a cold test: 0 = no block, 1 = analgesia (can feel touch but not cold) and 2 = anaesthesia (patient cannot feel touch) [21]. Motor block of the musculocutaneous, median, radial and ulnar nerves will be graded using a previously validated scale as either 0 = no block, 1 = paresis, 2 = paralysis [21]. Measurements will be continued until either the sensory block is adequate in the operative area distribution or a maximum of 20 minutes has elapsed at which point the block will be supplemented by targeted axillary or midhumeral supplementation as appropriate using ultrasound. If a BPB block fails despite supplementation, LA infiltration will be used. This will be recorded as a failed block. If intravenous opioid analgesia is required for operative site discomfort, the BPB will be recorded as a failed block. Anaesthetists performing ultrasound guided BPBs in this study will have been deemed competent to perform this technique by the principle investigator.

Patients in the Local Anaesthetic Infiltration group will receive infiltration of local anaesthetic into the surgical site by the operating surgeon under sterile conditions using a combination of 0.5% L-bupivicaine prior to incision and 1% lignocaine topically after the wound is opened. Maximum dose limits of 2 mg/kg for bupivicaine, and 3 mg/kg for lignocaine will be observed, recognising that these are additive.

Using the technique described above, at the points marked on the skin the internal diameters of the cephalic and basilic veins will again be measured. Because after a successful BPB patients will generally have an immobile, vasodilated, warm arm, blinding from this stage will be difficult. The average of three measurements at each position will again be calculated. Similarly, the arterial blood flow will be measured by Doppler ultrasound as described previously, and again the average of three measurements will be recorded. The veins and artery at the proposed surgical site will also be examined and mapped using ultrasound by the surgeon. The operating surgeon will use this information to decide on the final site for the fistula. Any deviation from the pre-operative plan based on anatomical information obtained using ultrasound at this stage will be recorded.

### Blinding

The allocation sequence will be accessed only when study data collection is complete or in any instance where unblinding of the study is thought to be essential in the provision of appropriate patient care. Due to the nature of the BPB and LA techniques, blinding of the patient, operating surgeon and observer measuring immediate post-anaesthetic flows is not possible. All further post-operative measurements, including the primary outcome, will be undertaken by a blinded observer.

### Intra-operative management

The anaesthetist looking after the patient in theatre will play no part in data analysis and will record the intra-operative proceedings as normal. The medications used to perform the two potential procedures will not be recorded on the anaesthetic chart. The patient’s participation in this study and the two possible anaesthetics that may have been received will be documented on the anaesthetic chart using a pre-made sticky label. Documentation of the anaesthetic procedures, including adverse events, will be kept in the patient’s case report file. The randomisation code may be accessed if deemed necessary in the provision of optimal patient care.

The patient will receive sedation if required and as directed by the anaesthetist. All medications, with the exception of the medications used to perform the BPB or local anaesthetic infiltration, will be detailed in the anaesthetic record.

All AVF procedures will be carried out at either the wrist, forearm or antecubital fossa. A radio-cephalic AVF will be fashioned through a longitudinal or equivalent incision and brachiocephalic AVFs fashioned using a horizontal or equivalent incision. Heparinized saline may be used to distend the vein. After sufficient mobilisation of the vein, an end-to-side or side-to-side anastamosis will be created using 6.0 or 7.0 non-absorbable suture. AVF patency peri-operatively will be confirmed by palpating for a thrill and listening for a bruit. If at this stage there is no evidence of patency, this will be recorded and surgical revision urgently undertaken if appropriate.

### Postoperative management

After surgery, patients will be taken to the recovery room and monitored according to standard hospital policy. Patient satisfaction and pain during the procedure will be assessed at this point. Patients will be discharged if they meet day surgery criteria as per hospital policy.

At a time period of one month, three months and one year, a blinded observer will examine AVF clinical patency by palpating for a thrill and auscultating for a bruit. At all these time points patients will be attending routine follow-up clinics or have commenced haemodialysis. Lack of both a thrill and a bruit will result in the fistula being defined as not patent. At the same time, brachial artery volume flow will be measured (as a surrogate for AVF flow) by a blinded observer using Doppler ultrasound as described previously. An average of three measurements will again be made in order to reduce both intra and inter-observer variation. Measurement of brachial artery flow is more reproducible than AVF flows and less prone to intraobserver variability [22]. The probe will be positioned at a distance of 2 to 5 cm cranial to the arterio-venous anastamosis. The point of measurement will be recorded and measurements at three months and one year made at the same position. A minimum of six weeks after AVF creation will be allowed to elapse before the first venipuncture for HD. The flow during the first cannulation of the fistula will be recorded by a blinded observer in the renal patient registry, and also at three months and one year if HD has been commenced at these time points. Finally, at each time point of one and three months and one year it will be noted by a blinded observer if there has been any infection of the AVF requiring antibiotics.

### Criteria for discontinuation

Every effort will be made to retain patients in the trial and to minimise withdrawals. However, any severe or life-threatening event will be sufficient to remove a patient from the study. Failure of either procedure is likely to result in the patient being given a general anaesthetic and, hence, being withdrawn from the study. Patients may request to be withdrawn from this study at any time. Reasons for withdrawal will be documented. Intention to treat and “as treated” analyses will be performed.

### Data collection

Data will be obtained from the anaesthetic record, recovery room observation chart, from clinical review in the post-operative period and from the electronic renal patient registry. Patient demographics, including age, gender, ethnicity, weight, body mass index and co-morbidities, as well as the surgeon, site of AVF (that is, distal or proximal arm), type of anastomosis (that is, end-to-side or side-to-side), duration of surgery, the type of anaesthesia used and fistula outcomes, will be recorded in the case report form. The researcher will be blinded to the technique used. Data will be anonymised. Case report files will be archived in a locked facility for a period of five years.

### Sample size and statistical considerations

Mean AVF failure after formation is reported to lie within the range 25 to 35% [4]. From our own retrospective audit data of patients receiving an ultrasound guided BPB over a two-year period, patients who had received BPB had an AVF survival rate of 93% compared with 52% in patients who received local anaesthetic infiltration [23]. In order to calculate sample size, we made the following assumptions: type 1 error (α) was set at 0.05 and Type 2 error (β) at 0.8. Therefore, if we assume a standard 65% AVF success rate overall, and propose that success rates with the use of BPB will be around 85%, then 57 patients are required in each group. In order to account for attrition of around 10%, we aim to recruit 126 patients. It should be noted that whilst these audit data were significantly in favour of the BPB, the data were retrospective, with non-standardised endpoints and also suffered from all the standard limitations of a retrospective analysis.

The null hypothesis (H0) for this study is that the use of ultrasound guided brachial plexus block does not reduce AVF primary failure rate when compared with local anaesthetic infiltration.

The study will be performed using both intention to treat and “as treated” analysis. In the intention to treat analysis, data will still be analysed from patients where the block has failed (that is if local anaesthetic supplementation is required), or if the patient was unable to receive randomised treatment for any other reason. In the “as treated” analysis, only data from patients completing randomised treatment will be analysed.

Secondary data analyses will be carried out on all secondary outcomes. These will be compared between groups using *t*-test and Mann–Whitney, or Chi-squared tests as appropriate.

It is anticipated that recruitment for this study will take up to two years to complete if one to two patients are enrolled each week. Data collection for each patient will occur during the operative and immediate post-operative period, and at dialysis sessions and post-operative follow-up as directed by the vascular team for one year.

We recognise that while this study is powered for the primary outcome, it is not powered for all the secondary outcomes. Whilst other studies suggest that our study is in fact overpowered for the secondary outcome of flow rate, this may not confer a clinical benefit, unlike our primary outcome, which is the reason this outcome has been selected. Any secondary data we collect in this study will provide useful information for further studies looking specifically at these outcomes.

### Adverse event reporting and safety

All adverse events will be fully recorded in the medical records and on the study record forms and discussed at monthly safety meetings by at least two investigators. Adverse events will be monitored and followed up until satisfactory resolution or stabilization. If clinically indicated, the nature of the anaesthetic administered in the study may be revealed. After assessment of adverse events by the principal investigator for seriousness, causality, expectedness and severity, any serious adverse events (SAEs) will be reported to the main Research and Ethics Committee and the sponsor where in the opinion of the principal investigator the event was related (resulted from administration of any of the research procedures) and unexpected (not listed in the protocol as an expected outcome). SAEs will be reported using the National Research Ethics Service SAE report form for non-Clinical Trials of Investigational Medical Products.

The anaesthetics used in this trial are commonly and safely used for a wide variety of surgeries, although, as with all techniques, rare risks exist. Rare risks, however, are also associated with general anaesthesia and because these are increased in patients with end stage renal failure, we expect that all patients will benefit through avoidance of a general anaesthetic. Unlike the local infiltration group, patients in the BPB group are at risk of nerve damage. This is a well-recognised complication of regional anaesthesia. Rates of temporary nerve damage for peripheral nerve blocks are reported in some studies to be as high as three percent [24]. Reassuringly, in this large review of many prospective and retrospective case series, the authors found no evidence of permanent damage. The frequency reported in other large case series was less frequent [25]. Nevertheless, nerve damage remains one of the most concerning risks of regional nerve blocks. Whilst in some studies this can be balanced against the benefit of avoiding the rare risks of general anaesthesia, in our study both groups will avoid general anaesthesia. The risk of nerve damage may be reduced by using ultrasound to guide needle placement. Ultrasound allows direct visualisation of the target nerve, needle and local anaesthetic spread and can detect small intraneural injections, but not intrafascicular injection, which appears to be most harmful [26]. Whilst ultrasound use appears to improve block success [27,28], it has not yet been proven to reduce the incidence of nerve damage. Indeed, nerve damage may still occur whilst ultrasound is in use [29]. Ultrasound has been shown, however, to reduce vascular puncture and allow use of smaller local anaesthetic volumes, the combination of which is important in reducing the risk of systemic local anaesthetic toxicity, which is the other serious but rare complication of regional anaesthesia [28,30]. Any adverse events relating to the procedure will be recorded by staff performing the study and necessary investigations, treatment or follow-up arranged thereafter.

## Conclusions

The purpose of this trial is to investigate the hypothesis that long-term AVF patency is improved by a BPB compared to local anaesthetic infiltration as this has not yet been demonstrated in a large randomised controlled trial. Reducing the AVF failure rate is of significant benefit by increasing the number of patients able to commence HD when planned and reducing the number of “redo” procedures. This has clear financial benefits and also reduces the inconvenience to patients and the risks of further AVF surgery. If, however, our null hypothesis is correct, this finding would still provide useful information. LA infiltration is simpler, less time consuming and avoids the rare risks of brachial plexus blocks. LA infiltration also bypasses the financial and practical implications of requiring an anaesthetist to undertake the block, and monitor the patient. Therefore, either a positive or negative result will help inform future anaesthetic practice regarding the surgical creation of arteriovenous fistulae.

## Abbreviations

AVF: Arteriovenous fisutla; BPB: Brachial plexus block; CKD: Chronic kidney disease; H0: Null hypothesis; HD: Haemodialysis; LA: Local anaesthetic; RRT: Renal replacement therapy; SAE: Serious adverse event.

## Competing interests

The authors declare that they have no competing interests.

## Authors’ contributions

AM and MC conceived of the study. RK, AM and MC designed the study. RK wrote the protocol. AM, EA, JK and MC reviewed and optimised the protocol. EA is responsible for communicating with the governing bodies (West of Scotland Research Ethics Committee, NHS Research and Development and NHS Finance Departments). RK, EA, JK, MC and AM have applied for funding for the study. MC is the principle investigator for the study. All authors have read and approved of the final version of the manuscript.
